# Predictors of Parental Acceptance towards Contemporary Behavior Management Techniques Used in Pediatric Dentistry: A Preliminary Study on Turkish Population

**DOI:** 10.3390/children10101592

**Published:** 2023-09-24

**Authors:** Merve Candan, Elifcan Kutlu, İmran Gökçen Yilmaz Karaman

**Affiliations:** 1 Department of Pedodontics, Faculty of Dentistry, Eskişehir Osmangazi University, Eskişehir 26040, Turkey; 2 Department of Psychiatry, Faculty of Medicine, Eskişehir Osmangazi University, Eskişehir 26040, Turkey; imrangokcen.yilmazkaraman@ogu.edu.tr

**Keywords:** pediatric dentistry, behavior management techniques, parental acceptance, children, dental treatment

## Abstract

Background: Parents, who make the final decision regarding their child’s treatment, play a significant role in their dental care. Parental approval is important for each stage of treatment and may affect the physician’s approach to the treatment. Therefore, it is essential for pediatric dentists to comprehend which behavior management techniques (BMTs) are acceptable to parents and to identify the factors that influence their acceptability. Methods: Parents who were bringing their children for dental treatment answered a survey on the acceptance of twenty contemporary BMTs. The sociodemographic data of the parents and the age, gender, and Frankl behavior rating scale (Frankl) scores of their children were recorded. Parents were asked to indicate their level of acceptance of each BMT. Binary logistic regression analysis was designed to see the predictors of “accepted” and “declined” answers. Results: According to acceptance frequencies, the most accepted technique was communication and communicative guidance. The most declined technique was parental absence. The child’s age, gender, Frankl score, parental educational status, and parent type predict the acceptability of some BMTs. Conclusion: This study revealed that pediatric dentists must consider parent and child factors when selecting BMTs for children. We believe that this study can provide a basis for determining which factors pediatric dentists should consider when selecting individualized BMTs.

## 1. Introduction

The child’s refusal to cooperate during dental treatment is a circumstance that complicates dental procedures. Numerous techniques for managing the child’s behavior have been devised to ensure treatment compliance. Although the American Academy of Pediatric Dentistry’s (AAPD) current guideline contains a variety of behavior management strategies, researchers are still looking for strategies that are acceptable to parents, effectively lower fear/anxiety, and change the child’s attitude during dental treatment [1,2,3].

The development and use of an individualized behavior management technique (BMT) based on the treatment needs of the pediatric patient is essential for the success of the treatments applied by pediatric dentists to children [4]. Behavioral guidance is an ongoing process that employs pharmacological and non-pharmacological techniques ranging from basic to advanced. Each BMT option should be evaluated and selected based on indications, contraindications, and precautions [1]. The vast majority of current techniques for behavior guidance are applicable to nearly all children. However, there may be a few exceptions. For example, animal-assisted therapy cannot be administered to children who fear or are allergic to animals. Cooperative children do not require protective stabilization, nitrous oxide/oxygen inhalation, sedation, or general anesthesia during dental procedures [1]. Predisposing medical conditions such as acute upper respiratory tract infections, cobalamin (vitamin B-12) deficiency, or allergy are contraindications to nitrous oxide/oxygen inhalation [1,5,6].

Dental anxiety/fear in children can affect their approach to dental treatment by reflecting on their behavior [7]. Additionally, pediatric patients’ responses to dental treatment vary. Variation in a child’s responses to dental treatment may be attributable to many factors such as age, maturity, temperament, prior dental experience, family history, culture, oral health status, parent’s dental anxiety, parenting style, and dentist’s communication and behavior [1,8,9,10]. In order to evaluate all these variables and provide influential behavioral guidance during the treatment of pediatric patients, pediatric dentists should also be able to communicate accurately and effectively with parents. Numerous factors play a significant role in the success of treatment and the selection of BMT, such as the sociocultural differences between the parents who make the final decision about the treatment of the children and their expectations from the dental treatment of their children [11,12].

Over time, some child BMTs have been reevaluated, while others have been abandoned. In the past, it has been reported that one of the BMTs, the hand-over-mouth technique (HOMT), has long-term benefits for the relationship between the child and the dentist [13]. However, according to a study conducted in 2001, the number of postdoctoral training programs advocating the use of the HOMT has decreased significantly due to uncertainty regarding the psychological consequences associated with the technique [14]. Lastly, HOMT is not included in the current AAPD guidelines [1]. However, in contemporary pediatric dentistry, new BMTs are being developed to reduce dental anxiety and increase treatment compliance. Although they are not yet included in the current AAPD guideline, the effects of many tools and methods, such as mobile applications and virtual reality headsets, on reducing dental anxiety in children have recently been determined [3,15].

In selecting the BMTs that pediatric dentists will use on their patients, the psychological side effects of the technique, the benefit-to-risk ratios associated with its use, especially for pharmacological BMTs, and parental acceptance are crucial considerations [1,14]. Although other evaluations are left to the discretion of the pediatric dentist, it is not appropriate to use BMT if the parents do not consent to its use [16]. It is crucial for pediatric dentists to understand which BMTs are still acceptable to parents and to identify factors that influence their acceptability. Recently, numerous studies have investigated whether or not parents accept BMTs [4,17,18,19,20].

As it is believed that a variety of factors may influence the decision of parents to accept a BMT, the purpose of the present study was to determine the most preferred and rejected BMTs by parents and some predictors of the current BMTs in the AAPD guidelines among Turkish parents. We hypothesize that (1) the child’s compliance with dental treatment would affect the acceptance of several BMTs, and (2) the parent’s and the child’s sociodemographic specifics would affect the acceptance of several BMTs.

## 2. Materials and Methods

The ethical approval for the current study was received from the Non-Interventional Clinical Research Ethics Committee of Eskişehir Osmangazi University (Approval Date/Number: 25 June 2023/25). The present study was conducted following the Helsinki Declaration [21]. Parents who consented to participate were given written and verbal information about the study’s purpose and nature.

The study was conducted between 26 July and 15 August 2023, following the ethics committee’s decision. The target population of the study consisted of 136 parents who brought their children to the Eskişehir Osmangazi University Department of Pediatric Dentistry and volunteered to participate in the study. To standardize the parents, parents of children with special needs requirements and parents who brought their children for emergency treatments were excluded.

### 2.1. The Study Questionnaire

The data collection form was a self-administered paper-and-pencil questionnaire in the Turkish language. It had three parts. The first part aimed to learn the parents’ and children’s demographic information. The second part asked the parent about their child’s level of compliance during previous dental treatment. The Frankl Behavior Rating Scale was used to examine the child’s treatment compliance. The score on this scale ranges from 1 (extremely uncooperative) to 4 (extremely cooperative) [22]. Parents stated their child’s cooperation according to the Frankl Behavior Rating Scale.

The third part was about parental acceptance of BMTs. Techniques were explained with written definitions of BMTs according to the current clinical guidelines of the AAPD on BMTs [1] in the survey, similar to a previous study [4]. Definitions of BMTs were summarized in one sentence each by M.C., in Turkish. Original descriptions of the BMTs and the Turkish summaries were sent to three senior dentist academicians for review. After revisions, M.C. performed a pilot study on ten parents to see if the questionnaire was comprehensible. Since the feedback was positive, the third part of the study questionnaire took its final form. The parents were asked to state their level of acceptance of each BMT. The acceptability rating was determined as “I accept”, “I decline”, and “I hesitate”.

### 2.2. Procedure

While their child was being treated, parents completed the questionnaire in the treatment room or waiting room. Parents were given time to complete the survey on their own. E.K. was present to explain BMTs in case the parents had questions about them. Only one illiterate parent was read the survey questions by E.K. When the parents requested additional information about any BMT, E.K. explained the relevant BMT.

### 2.3. Statistical Analysis

IBM SPSS (Statistical Package for the Social Sciences) Version 26 was utilized for the present study. Categorical variables were presented as frequency and percentage. Continuous variables were presented with means and standard deviations. The Kolmogorov–Smirnov test tested the normal distribution of the data. The relationship between two continuous variables was tested with the Spearman correlation test. The median values of two independent samples were compared with the Mann–Whitney U test. A binary logistic regression analysis with the Backward Wald method was utilized to determine the parent’s BMT preference predictors. A statistically significant *p*-value was set at 0.05.

## 3. Results

One hundred thirty-six parents participated in the present study. The characteristics of the participants are presented in Table 1. The children’s characteristics are listed in Table 2. The children’s age and Frankl score were associated (ρ = 0.439, *p* < 0.001). Girls and boys were not different regarding Frankl scores (U = 2276.000, *p* = 0.958). 

Table 3 summarizes parents’ preferences for BMTs. The first most popular quartile (>124 participants) was calculated according to acceptance frequencies. The most popular BMTs were: (1) Communication and communicative guidance; (2) Positive reinforcement and descriptive praise; (3) Enhancing control; (4) Tell-show-do; and (5) Ask-tell-ask.

The most frequently rejected techniques were determined in the first quartile (>30 participants). The most rejected techniques were (1) Parental absence, (2) General anesthesia, (3) Protective stabilization, (4) Animal-assisted therapy, and (5) Voice control.

Binary logistic regression analysis was designed to see the predictors of “accepted” and “declined” answers. ‘Communication and communicative guidance’ and ‘Tell-show-do’ techniques were not included since they have no “declined” answers. Variables of children’s age, gender, Frankl score, parent type, and parent educational level were selected to test their predictivity. For every BMT, models were tested with the “Backward Wald” method. To evaluate model performance, each model was tested with the Omnibus test and the Hosmer-Lemeshow test. Models with the Omnibus test’s *p*-value less than 0.05 and the Hosmer-Lemeshow test’s *p*-value higher than 0.05 were accepted. Only models with statistically significant predictors are listed in Table 4.

The findings that follow are the outcomes of binary logistic regression models (see Table 4 for statistical analysis results):

- A higher Frankl score predicts the acceptability of the ‘Voice control’ technique;
- A higher Frankl score predicts the acceptability of the ‘Nonverbal communication’ technique;
- A lower education level predicts the acceptability of the ‘Nonverbal communication’ technique;
- A higher Frankl score predicts the acceptability of the ‘Memory restructuring’ technique;
- A lower child’s age predicts the acceptability of the ‘Memory restructuring’ technique;
- A higher child’s age predicts the acceptability of the ‘Parental absence’ technique;
- The acceptability of the ‘Nitrous oxide/oxygen inhalation and Sedation’ technique can be predicted by the child’s gender (having a son rather than a daughter) and a higher level of parental education;
- Having a son rather than a daughter predicts the acceptability of the ‘Protective stabilization’ technique;
- A lower child’s age can predict the acceptability of the ‘Protective stabilization’ technique. Additionally, being the mother versus the father predicts the acceptability of the technique.

## 4. Discussion

The present study determined that parents’ acceptance of some BMTs was predicted by the child’s compliance with dental treatment and the parent’s and the child’s sociodemographic specifics, paralleling the present study’s hypotheses.

Pediatric dentists’ BMT preferences can be influenced by their knowledge of children’s attitudes toward dental care and their parents’ acceptance rates of BMTs. The acceptability of BMTs among parents for their children’s dental care depends on the parent’s socioeconomic and educational status, ethnic and cultural backgrounds, parenting style, and prior negative dental experiences [4,18,23,24]. In addition, factors related to the child, such as the child’s need for special health care, birth order, dental anxiety, level of understanding, and prior experience with BMTs, may also influence parental acceptance decisions of BMTs [23,25,26,27].

It is known that children’s behavior is affected by numerous factors, including their parents [11]. Dental anxiety is among the most significant factors influencing a child’s attitude toward dental treatments [8]. Knowing or determining whether children seeking treatment have dental anxiety enables pediatric dentists to be prepared for the possible reactions children may have to treatment and to take measures to reduce their anxiety and fear [28]. Various scales are used to evaluate a child’s behavior and treatment compliance during dental care. The Frankl Behavior Rating Scale [22] is one of the most frequently utilized behavioral assessment scales in daily life and pediatric dentistry research [29]. Researchers frequently employ this scale to evaluate a child’s response to various variables [3,7,10,11]. In accordance with this, the Frankl Behavior Rating Scale, a behavioral observation instrument, was utilized to assess treatment compliance in the current study.

According to a study, no statistically significant difference existed between distinct explanation methods (written, verbal, and video film screening) and the parental acceptance of BMT [30]. Hence, in the current study, the techniques were explained by definition and informative written notes according to the AAPD’s contemporary ‘Behavior Guidance for the Pediatric Dental Patient’ guideline, revised in 2020 [1].

Communicative management and appropriate commands are universally utilized in pediatric dentistry on cooperative and uncooperative children [1]. This technique is the most preferred BMT by parents in the present study. However, parental absence was the most frequently rejected technique. Similarly, studies determined that most parents preferred to stay with their children during dental treatments [23,31]. Despite parental preferences in this direction, it has been determined that the parental presence/absence technique does not affect the dental anxiety and treatment compliance of 5-year-old children [32]. In the present study, animal-assisted therapy was among the top five most rejected techniques. A current meta-analysis found no evidence for or against the effectiveness of this technique in reducing anxiety in children aged 5 to 11 years [33]. One of the reasons why this technique is not preferred may be that children fear animals. Therefore, in the present study, parents may not accept animal-assisted therapy because they do not believe it will increase their child’s treatment adherence. Moreover, the order of preference for some of the basic and advanced BMTs accepted in our study was similar to prior investigations [20,34].

According to a previous study, there is no correlation between parental dental anxiety and the acceptance of any specific BMT (3). However, children’s dental fear/anxiety is a condition that impedes treatment compliance [10]. The present study found that higher Frankl scores predicted the acceptability of ‘voice control’, ‘nonverbal communication’, and ‘memory restructuring’ techniques.

Many parents believe that their presence during a child’s treatment will increase the child’s stability and comfort, and they may want to hold their child’s hands [35]. In contrast, a previous study found that the parental presence/absence technique was not more effective than other non-pharmacological basic BMTs (5). However, in the present study, the parental absence technique was determined to be the technique most frequently denied, and the child’s age was a predictor of the technique’s acceptability. A higher child’s age predicts the acceptability of the parental absence technique. That may be due to parents’ belief that they can support their small kids during treatment or their desire to observe them.

It is known that parental type influences a child’s attitude toward dental treatment [11]. In the present study, motherhood predicts acceptance of the protective stabilization technique. According to a previous study, mothers evaluated the protective stabilization technique as beneficial despite its emotional distress (6).

Children frequently cannot cooperate with the dentist during dental treatments due to a lack of emotional or psychological maturity or a physical, medical, or mental disability [1]. In such a situation, advanced behavior guidance may be the technique of choice for these children. The advanced BMTs include protective stabilization, sedation, and general anesthesia [1]. However, parental approval must be obtained before implementing these techniques. Before obtaining parental informed consent, discussing the benefits, risks, and alternative options of advanced BMTs is necessary. According to a study conducted in Germany, parents were more willing to approve advanced BMT in emergencies, and nitrous oxide sedation was the most preferred technique overall [4].

In specific situations, such as dental emergencies of brief duration, protective stabilization techniques may be used if the child does not cooperate adequately for dental treatment. The gender of the physician, the practice environment, the region, and the perception of parental acceptance are essential factors in the acceptance and use of the protective stabilization technique [36]. Protective stabilization may not always be accepted by parents, who may be more accepting of pharmacologic BMTs [1,12]. The current study’s findings suggest that a child’s lower age can predict the acceptability of the protective stabilization technique. Additionally, having a son rather than a daughter and motherhood (parent type) predict the acceptability of the technique. According to the present study, children’s adherence to dental treatment is related to a child’s age. The fact that most of the caregivers of children are mothers and that it is a quick and inexpensive way to solve the existing dental problems of their young children who have insufficient compliance with dental treatment may have affected the technical acceptability. However, the adverse psychological side effects that may arise from this technique should be considered [37].

Sedation is a technique that can be used effectively and safely with patients who are uncooperative due to a lack of emotional or psychological maturity or a physical, medical, or mental disability [1]. The high incidence of caries, parental expectations, and challenging child behaviors support the need for sedation [38]. A report indicates that parents consider sedative techniques safe [39]. However, in a study conducted among Asian, Caucasian, and Hispanic parents, ethnicity created a significant difference between groups in conscious sedation preference (*p* = 0.047), with Asian parents preferring this technique the least [40]. In the present study conducted with Turkish parents, only half of the parents accepted the ‘Nitrous oxide/oxygen inhalation and Sedation’ technique. Also, the acceptability of the technique can be predicted by the child’s gender (having a son rather than a daughter) and a higher level of parental education. This situation may be because education can increase the research rate and access to accurate information that will eliminate prejudices about medication applications.

According to the present study, the child’s gender (having a son rather than a daughter) predicts protective stabilization and sedation techniques. That may be because boys are more active than girls during infancy, childhood, and adolescence [41,42].

In severe clinical circumstances, such as early childhood caries or dental surgery, general anesthesia may be necessary even when the child’s cooperation is sufficient [1,43]. Thanks to their BMT preferences, parents can have the opportunity to choose whether their child’s dental treatment will be performed under local or general anesthesia. The general anesthesia technique was Turkish parents’ second most rejected BMT technique after parental absence, according to the present study. Therefore, it can be inferred that parents have apprehensions regarding general anesthesia.

The AAPD guideline classifies protective stabilization, sedation, and general anesthesia as advanced behavior guidance, while all other BMTs in the present study are classified as essential behavior guidance [1]. A meta-analysis conducted in 2022 revealed that parents were more likely to accept basic BMTs than advanced BMTs [27]. Similarly, this study determined that the acceptance rates of basic BMTs were higher than those of advanced BMTs, except for animal-assisted therapy and parental absence techniques.

Pediatric dentists need to consider the ethnic/cultural differences of parents in order to provide higher-quality treatment for pediatric patients [40]. Studies found that parents of different ethnicities embrace different BMTs differently [12,40]. Therefore, we believe that Turkish cultural characteristics may also influence the findings of the current study. The presence of only Turkish parents in the study can be considered a limitation. Although this study evaluated numerous parent and child variables in BMT selection, other limitations include not requesting the child’s dental history in the questionnaire, the parent’s statement of the child’s level of compliance with dental treatment, and the exclusion of a substantial number of other psychological and environmental variables.

In conclusion, parental acceptance of BMTs for their children is influenced by several factors associated with the parents and the children. According to the present study, the child’s age, gender, compliance status with dental treatment, parental educational status, and parent type predict the Turkish parent’s acceptability of various BMTs. All parents accepted the ‘Communication and communicative guidance’ and ‘Tell-show-do’ techniques. Parental absence and general anesthesia were determined to be the two most rejected techniques. The acceptance rates of basic BMTs were higher than those of advanced BMTs, except for animal-assisted therapy and parental absence techniques. Pediatric dentists should consider parental and child variables when selecting BMTs for children. This study can provide a basis for determining which factors pediatric dentists should consider when selecting individualized BMTs for children.

## Figures and Tables

**Table 1:** The characteristics of the participants (*n* = 136).

		*n*	*%*
**Parent type**	*Mother*	99	72.8
	*Father*	37	27.2
**Age**	*18–30*	14	10.3
	*31–40*	75	55.15
	*41–50*	45	33.1
	*50+*	2	1.5
**Education level**	*Analphabet*	1	0.7
	*Primary school*	21	15.4
	*High school*	49	36
	*University graduate*	53	39
	*Postgraduate*	12	8.8
**Employment**	*Yes*	77	56.6
	*No*	59	43.4

**Table 2:** The characteristics of the children (*n* = 136).

		Mean ± SD	
Child’s age		7.88 ± 2.70	
		*n*	%
**Child’s gender**	*Girl*	61	44.9
	*Boy*	75	55.1
**Frankl’s Behavior Rating Scale Score**	*1*	25	18.4
	*2*	39	28.7
	*3*	47	34.6
	*4*	25	18.4

**Table 3:** Parental preferences regarding behavior management techniques (*n* = 136).

		Accept	Hesitant	Decline
		*n* (%)	*n* (%)	*n* (%)
**1**	*Communication and communicative guidance*	134 (98.5%)	2 (1.5%)	0 (0%)
**2**	*Positive pre-visit imagery*	101 (74.3%)	28 (20.6%)	7 (5.1%)
**3**	*Direct observation*	70 (51.5%)	40 (29.4%)	26 (19.1%)
**4**	*Tell-show-do*	128 (94.1%)	8 (5.9%)	0 (0%)
**5**	*Ask-tell-ask*	126 (92.6%)	6 (4.4%)	4 (2.9%)
**6**	*Voice control*	72 (52.9%)	33 (24.3%)	31 (22.8%)
**7**	*Nonverbal communication*	82 (60.3%)	27 (19.19%)	27 (19.19%)
**8**	*Positive reinforcement and descriptive praise*	130 (95.6%)	5 (3.7%)	1 (0.7%)
**9**	*Distraction*	105 (77.2%)	19 (14%)	12 (8.8%)
**10**	*Memory restructuring*	114 (83.8%)	19 (14%)	3 (2.2%)
**11**	*Desensitization to dental setting and procedures*	71 (52.2%)	44 (32.4%)	21 (15.4%)
**12**	*Enhancing control*	130 (95.6%)	4 (2.9%)	2 (1.5%)
**13**	*Parental presence*	93 (68.4%)	33 (24.3%)	10 (7.4%)
**14**	*Parental absence*	32 (23.5%)	37 (27.2%)	67 (49.3%)
**15**	*Sensory-adapted dental environments*	118 (86.8%)	15 (11%)	3 (2.2%)
**16**	*Animal-assisted therapy*	52 (38.2%)	50 (36.8%)	34 (25%)
**17**	*Picture exchange communication system*	106 (77.9%)	27 (19.9%)	3 (2.2%)
**18**	*Nitrous oxide/oxygen inhalation and sedation*	68 (50%)	45 (33.1%)	23 (16.9%)
**19**	*Protective stabilization*	57 (41.9%)	32 (23.5%)	47 (34.6%)
**20**	*General anesthesia*	41 (30.1%)	31 (22.8%)	64 (47.1%)

**Table 4:** Binary logistic regression models with statistically significant predictors (*n* = 136).

	Omnibus Test	Hosmer - Lemeshow Test	Predictor	Odds Ratio	*p*
**Voice control**	χ^2^ = 7.952 *p* = 0.005	χ^2^ = 1.107 *p* = 0.575	Child’s Frankl score	1.914 [1.196–3.061]	0.007
**Nonverbal communication**	χ^2^ = 24.897 *p* < 0.001	χ^2^ = 5.136 *p* = 0.527	Child’s Frankl score	2.256 [1.330–3.826]	0.003
			Educational level	0.122 [0.036–0.413]	0.001
**Memory restructuring**	χ^2^ = 7.344 *p* = 0.025	χ^2^ = 5.736 *p* = 0.677	Child’s Frankl score	7.705 [1.026–57.840]	0.047
			Children’s age	0.561 [0.308–1.020]	0.058
**Parental absence**	χ^2^ = 12.704 *p* < 0.001	χ^2^ = 4.715 *p* = 0.581	Children’s age	1.348 [1.130–1.607]	0.001
**Nitrous oxide/oxygen inhalation and sedation**	χ^2^ = 9.312 *p* = 0.010	χ^2^ = 0.118 *p* = 0.913	Children’s gender	2.249 [0.794–6.368]	0.127
			Educational level	3.520 [1.260–9.830]	0.016
**Protective stabilization**	χ^2^ = 8.477 *p* = 0.037	χ^2^ = 13.205 *p* = 0.105	Children’s gender	2.373 [1.026–5.491]	0.043
			Children’s age	0.878 [0.749–1.029]	0.108
			Parent type	0.388 [0.151–1.002]	0.050

## Data Availability

The data presented in this study are available within this article.
